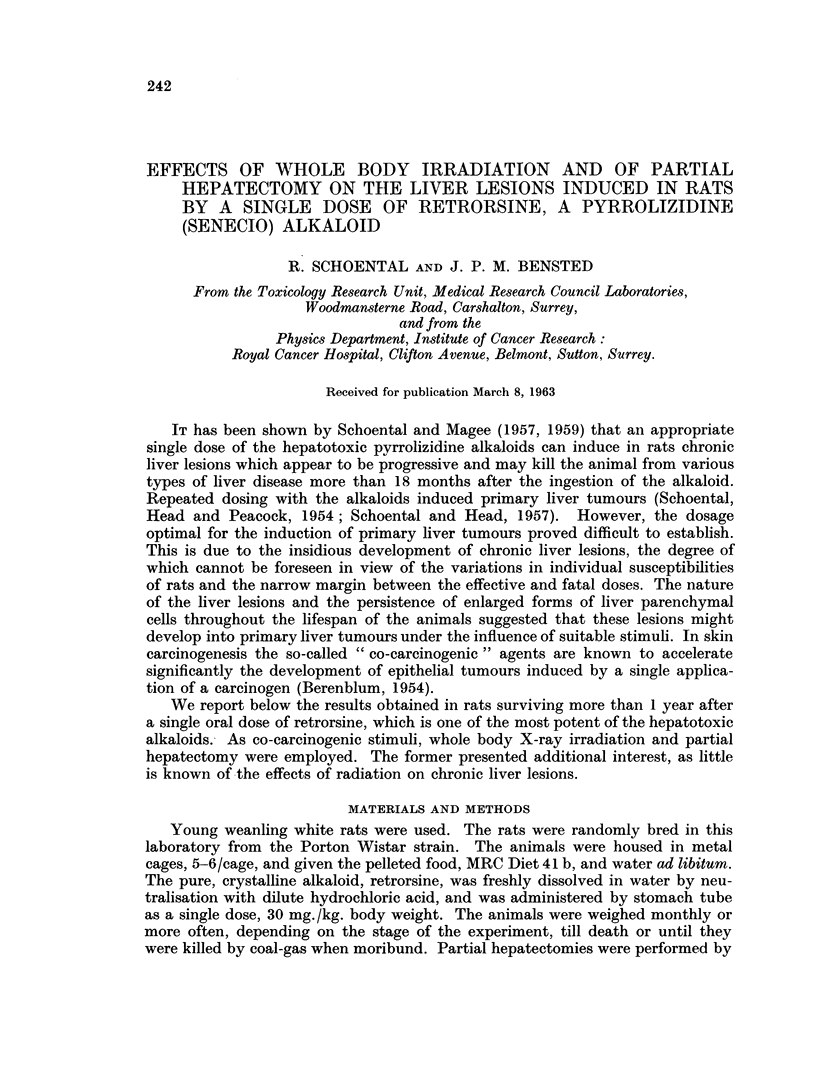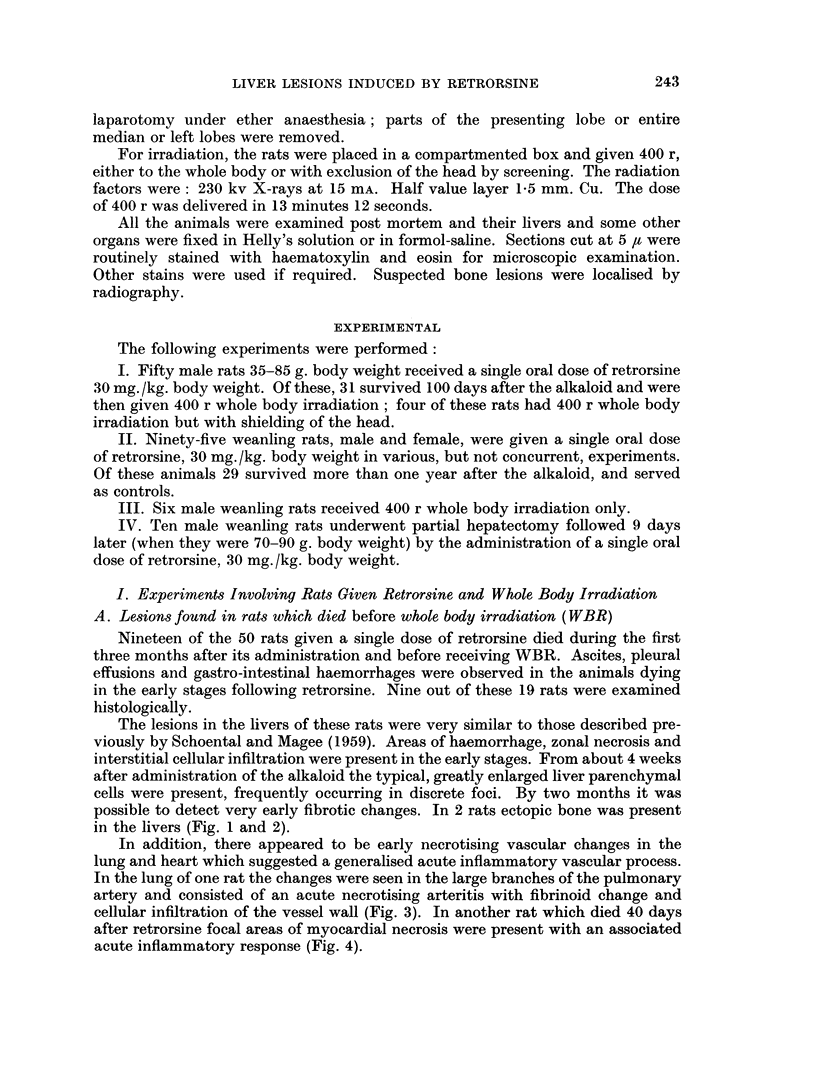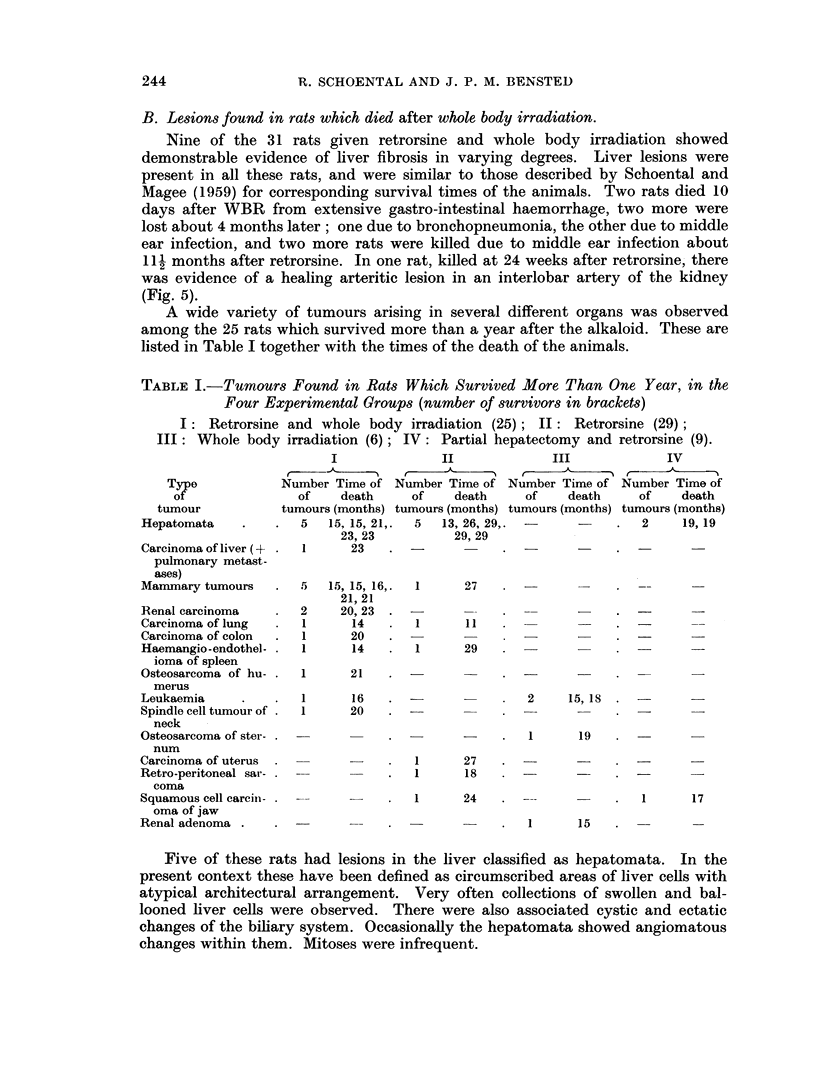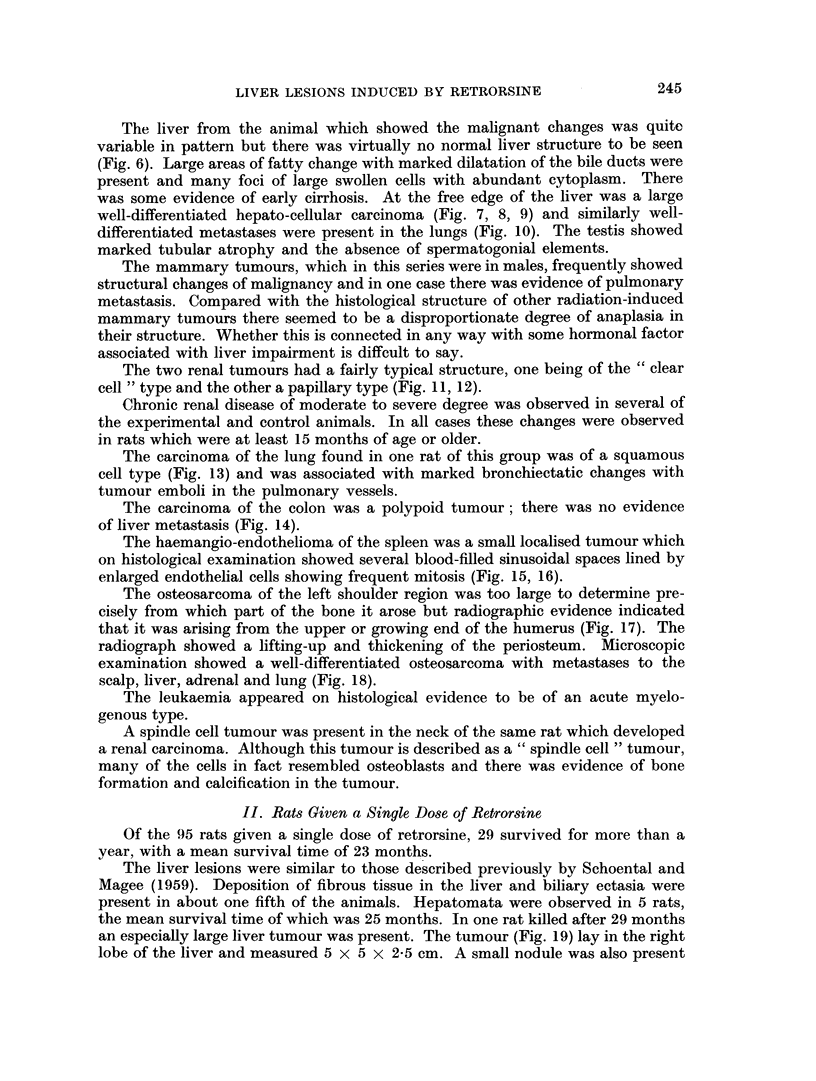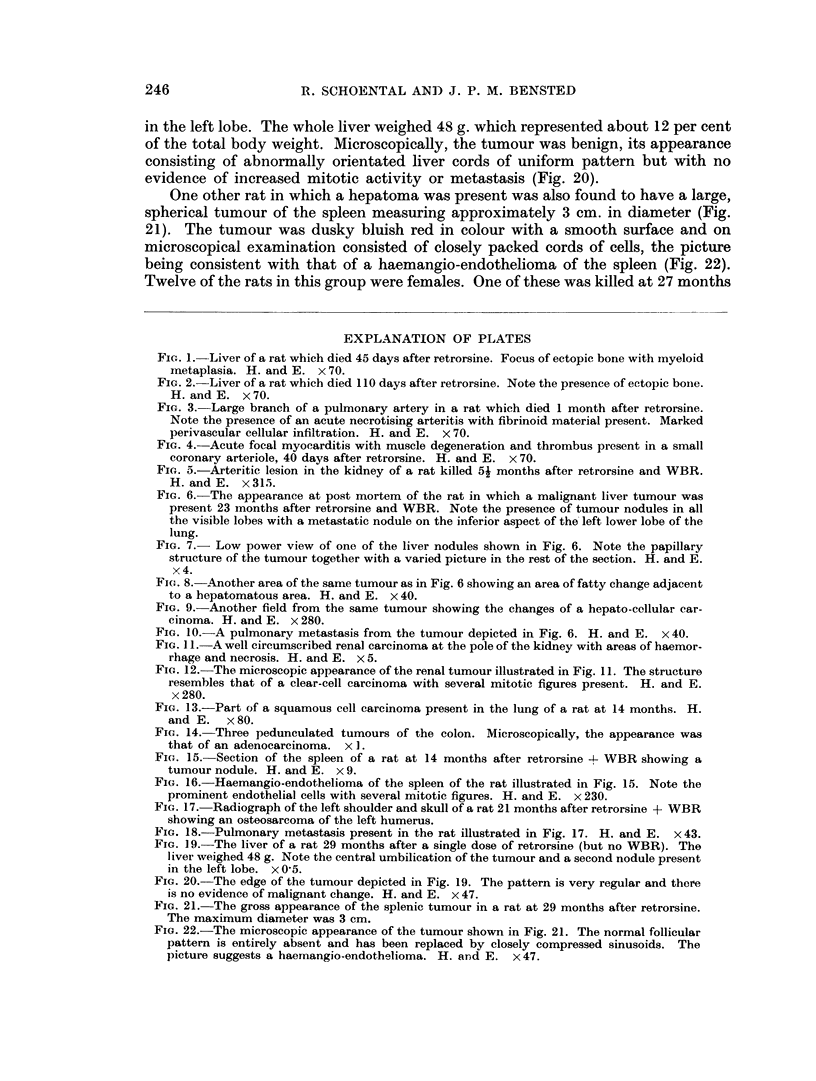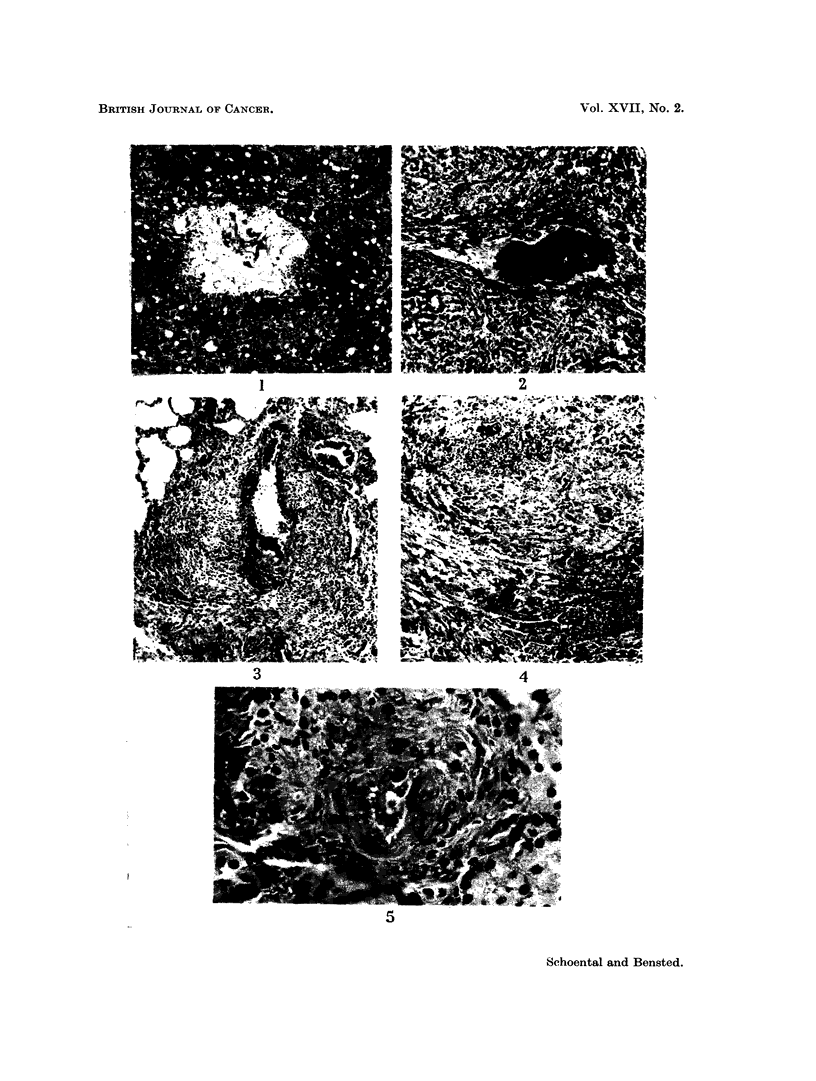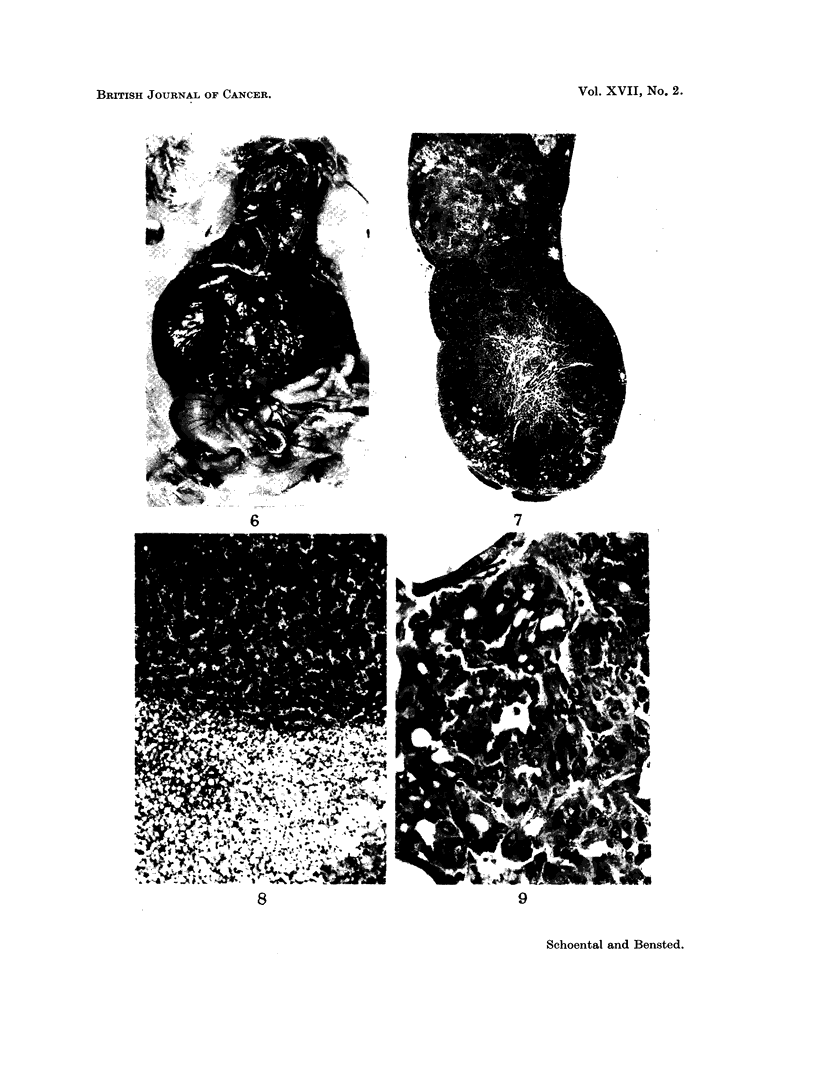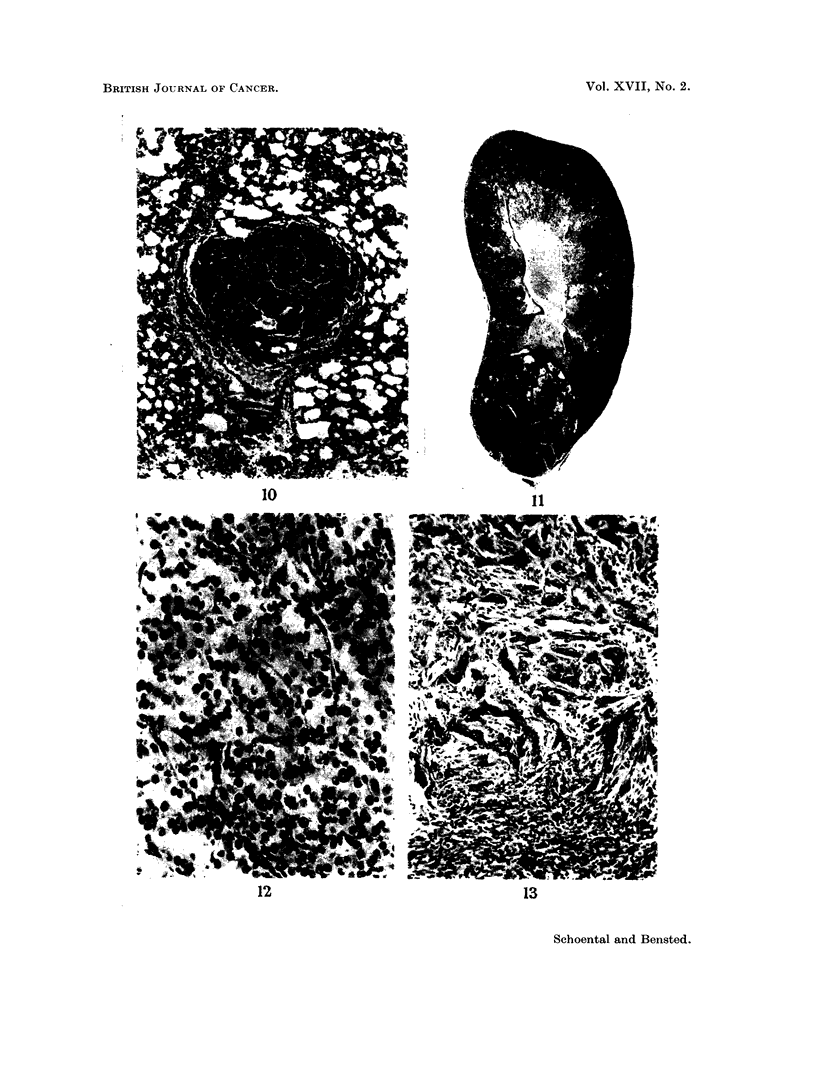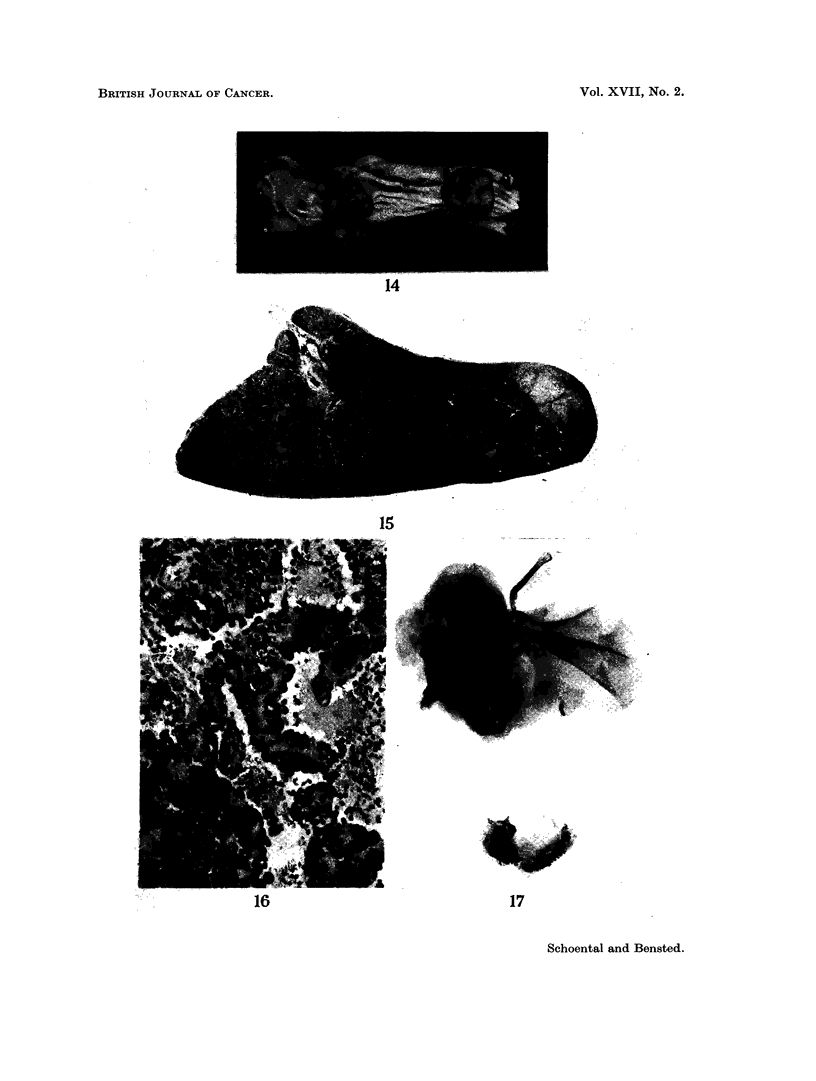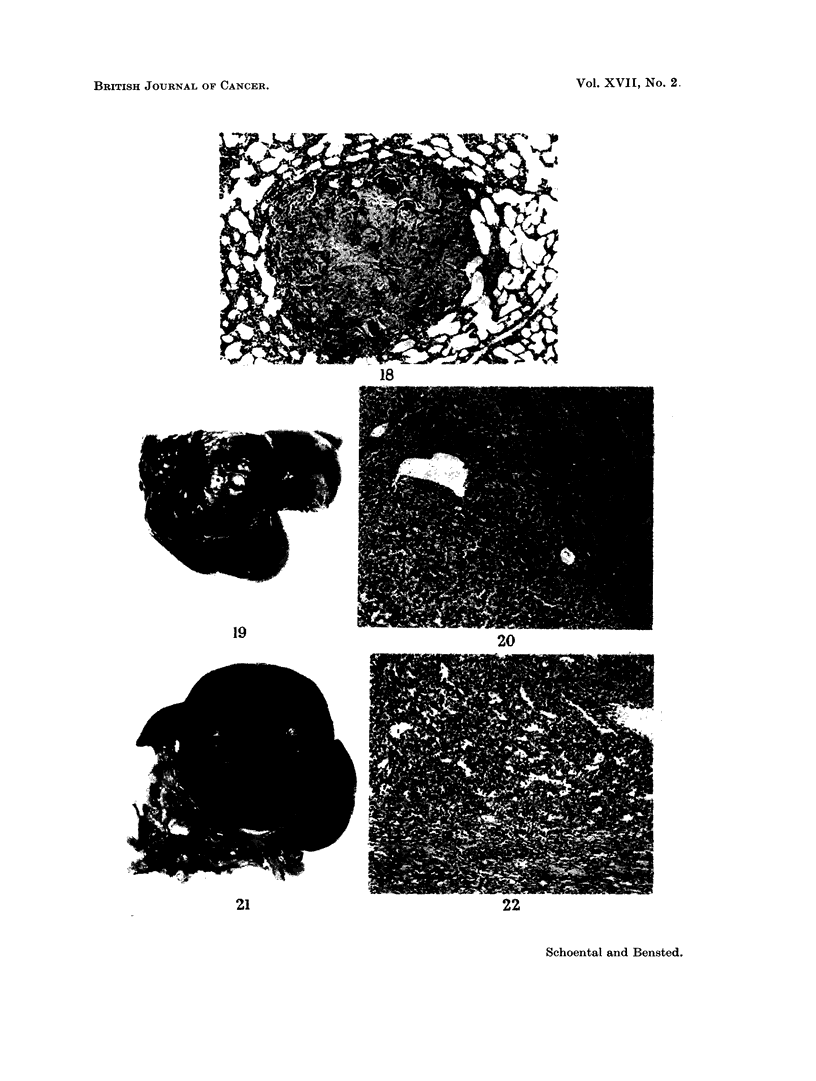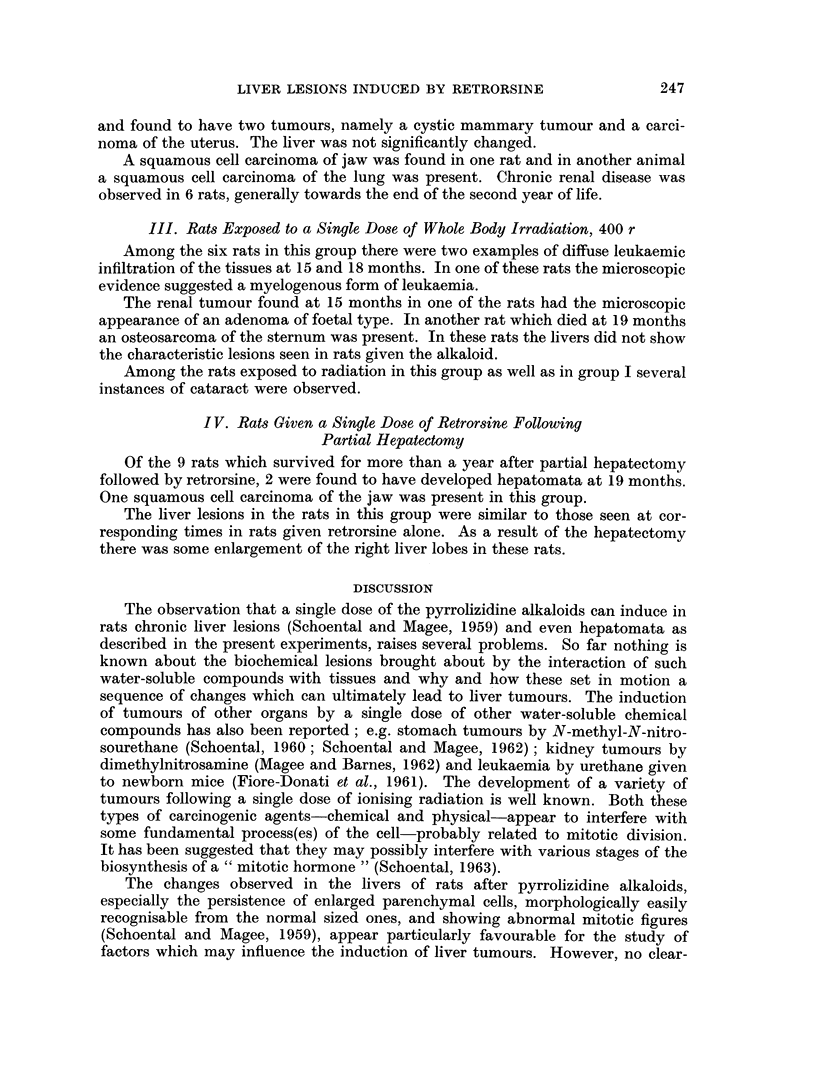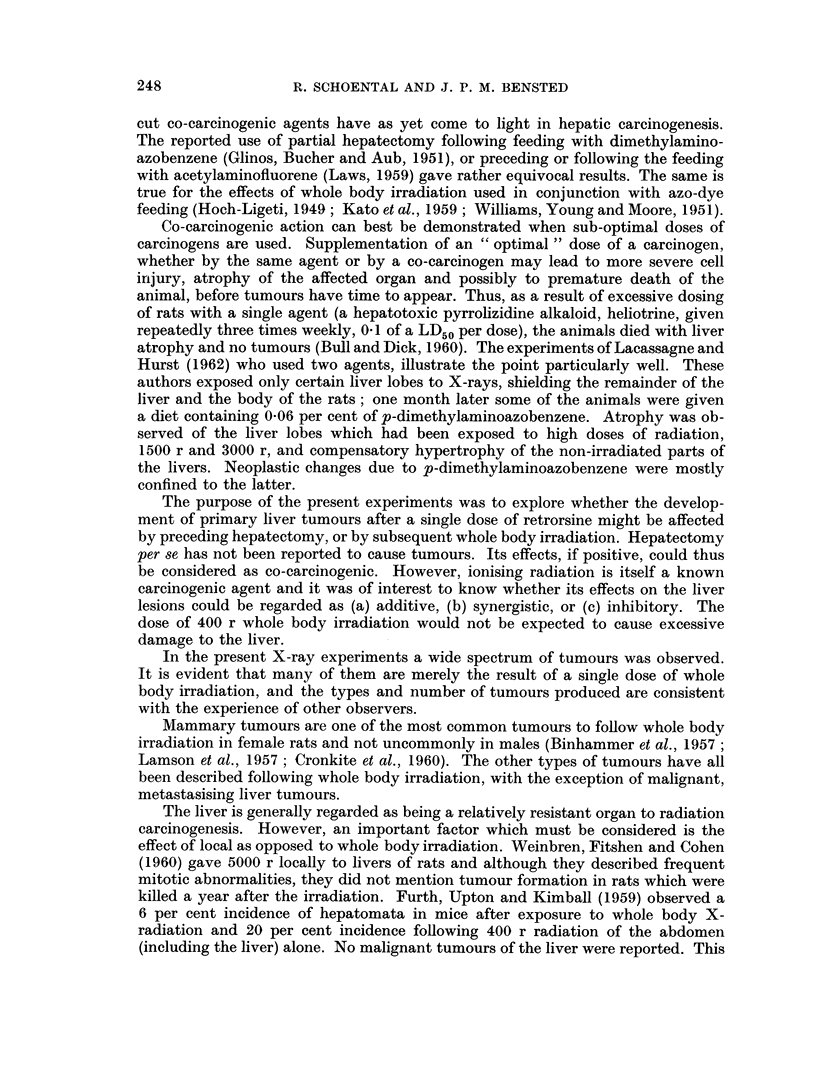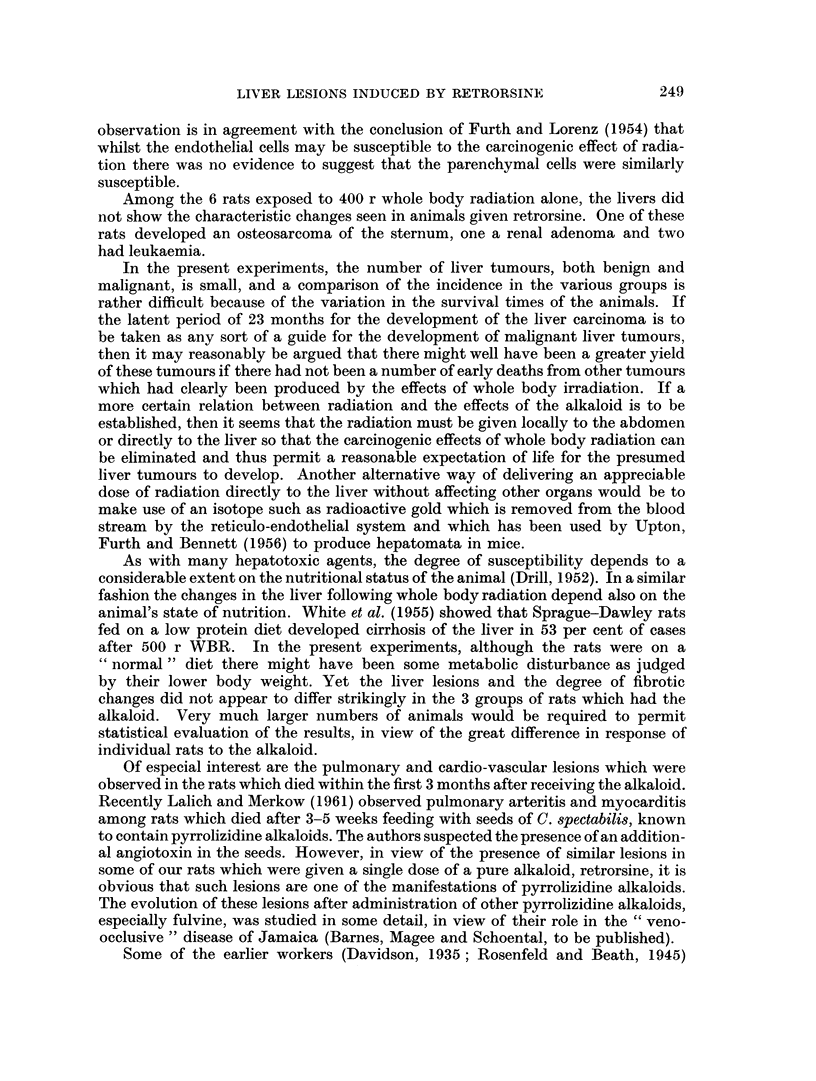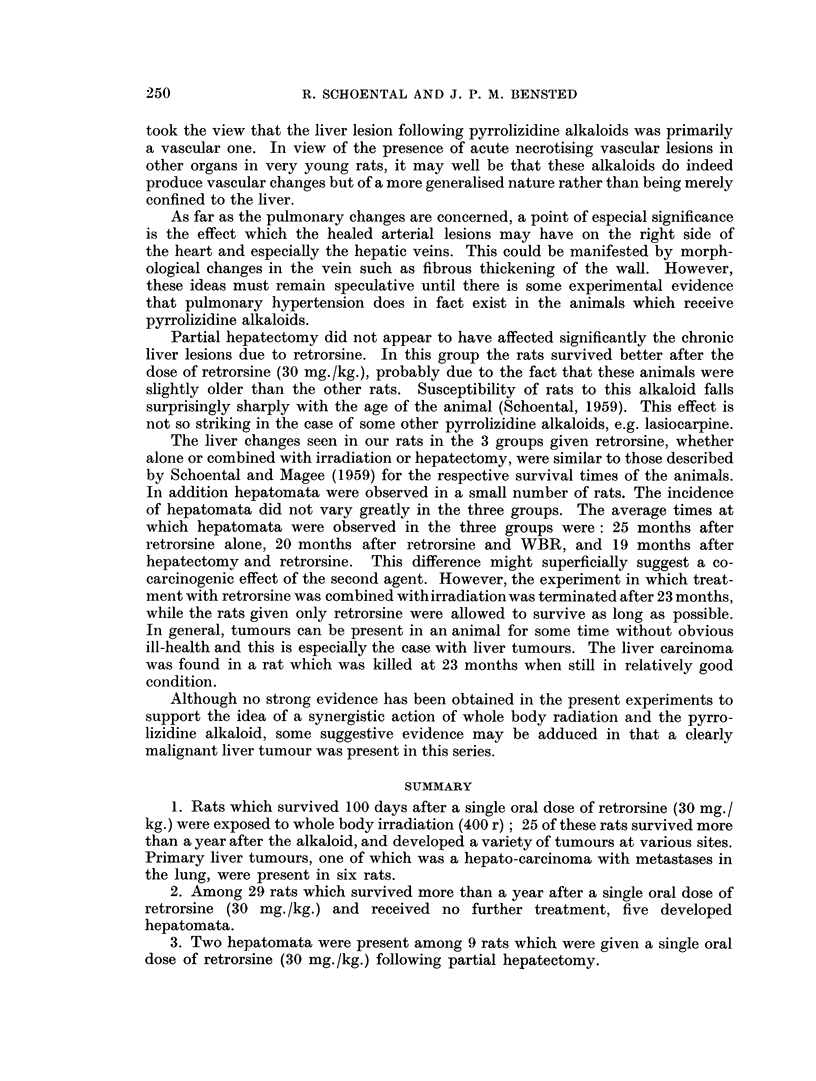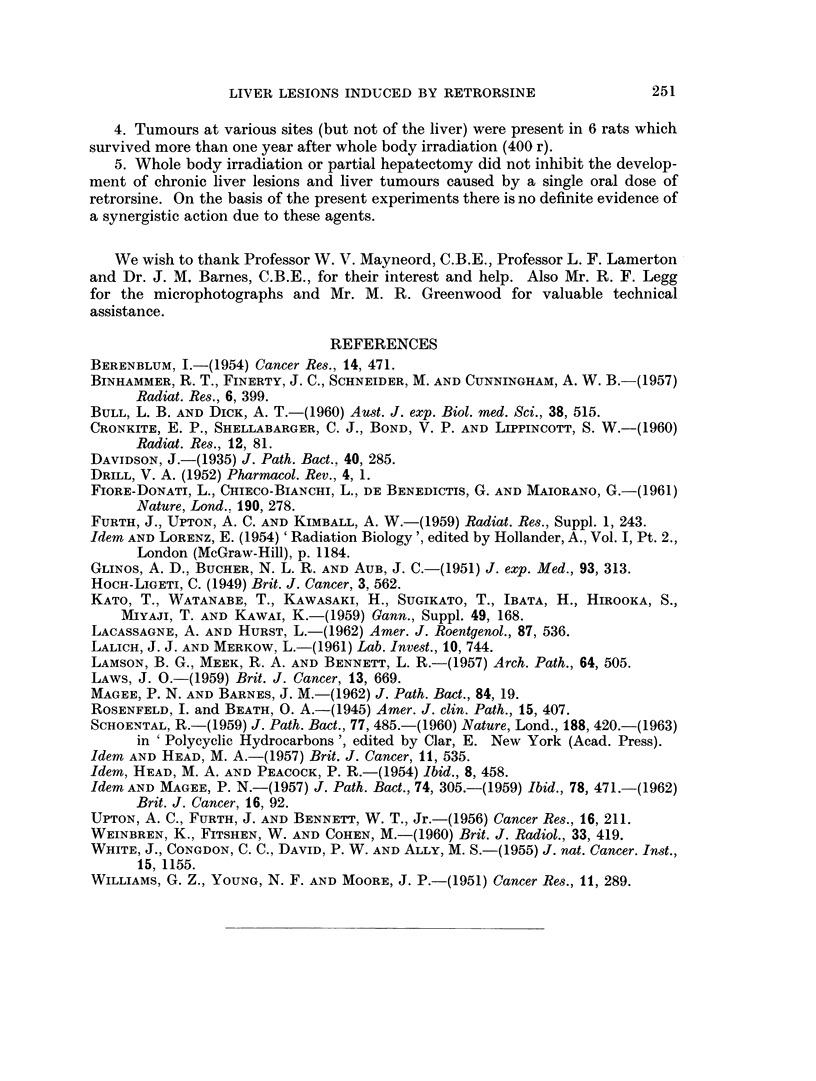# Effects of Whole Body Irradiation and of Partial Hepatectomy on the Liver Lesions Induced in Rats by a Single Dose of Retrorsine, a Pyrrolizidine (Senecio) Alkaloid

**DOI:** 10.1038/bjc.1963.34

**Published:** 1963-06

**Authors:** R. Schoental, J. P. M. Bensted

## Abstract

**Images:**


### EFFECTS OF WHOLE BODY IRRADIATION AND OF PARTIAL HEPATECTOMY ON THE LIVER LESIONS INDUCED IN RATS BY A SINGLE DOSE OF RETRORSINE, A PYRROLIZIDINE (SENECIO) ALKALOID

R. SCHOENTAL AND J. P. M. BENSTED

*From the Toxicology Research Unit, Medical Research Council Laboratories, Woodmansterne Road, Carshalton, Surrey, and from the Physics Department, Institute of Cancer Research : Royal Cancer Hospital, Clifton Avenue, Belmont, Sutton, Surrey.*

Received for publication March 8, 1963

It has been shown by Schoental and Magee (1957, 1959) that an appropriate single dose of the hepatotoxic pyrrolizidine alkaloids can induce in rats chronic liver lesions which appear to be progressive and may kill the animal from various types of liver disease more than 18 months after the ingestion of the alkaloid. Repeated dosing with the alkaloids induced primary liver tumours (Schoental, Head and Peacock, 1954 ; Schoental and Head, 1957). However, the dosage optimal for the induction of primary liver tumours proved difficult to establish. This is due to the insidious development of chronic liver lesions, the degree of which cannot be foreseen in view of the variations in individual susceptibilities of rats and the narrow margin between the effective and fatal doses. The nature of the liver lesions and the persistence of enlarged forms of liver parenchymal cells throughout the lifespan of the animals suggested that these lesions might develop into primary liver tumours under the influence of suitable stimuli. In skin carcinogenesis the so-called " co-carcinogenic " agents are known to accelerate significantly the development of epithelial tumours induced by a single application of a carcinogen (Berenblum, 1954).

We report below the results obtained in rats surviving more than 1 year after a single oral dose of retrorsine, which is one of the most potent of the hepatotoxic alkaloids. As co-carcinogenic stimuli, whole body X-ray irradiation and partial hepatectomy were employed. The former presented additional interest, as little is known of the effects of radiation on chronic liver lesions.

### MATERIALS AND METHODS

Young weanling white rats were used. The rats were randomly bred in this laboratory from the Porton Wistar strain. The animals were housed in metal cages, 5–6/cage, and given the pelleted food, MRC Diet 41 b, and water *ad libitum*. The pure, crystalline alkaloid, retrorsine, was freshly dissolved in water by neutralisation with dilute hydrochloric acid, and was administered by stomach tube as a single dose, 30 mg./kg. body weight. The animals were weighed monthly or more often, depending on the stage of the experiment, till death or until they were killed by coal-gas when moribund. Partial hepatectomies were performed by laparotomy under ether anaesthesia ; parts of the presenting lobe or entire median or left lobes were removed.

For irradiation, the rats were placed in a compartmented box and given 400 r, either to the whole body or with exclusion of the head by screening. The radiation factors were : 230 kv X-rays at 15 mA. Half value layer 1·5 mm. Cu. The dose of 400 r was delivered in 13 minutes 12 seconds.

All the animals were examined post mortem and their livers and some other organs were fixed in Helly's solution or in formol-saline. Sections cut at 5 $\mu$ were routinely stained with haematoxylin and eosin for microscopic examination. Other stains were used if required. Suspected bone lesions were localised by radiography.

### EXPERIMENTAL

The following experiments were performed :

I. Fifty male rats 35–85 g. body weight received a single oral dose of retrorsine 30 mg./kg. body weight. Of these, 31 survived 100 days after the alkaloid and were then given 400 r whole body irradiation ; four of these rats had 400 r whole body irradiation but with shielding of the head.

II. Ninety-five weanling rats, male and female, were given a single oral dose of retrorsine, 30 mg./kg. body weight in various, but not concurrent, experiments. Of these animals 29 survived more than one year after the alkaloid, and served as controls.

III. Six male weanling rats received 400 r whole body irradiation only.

IV. Ten male weanling rats underwent partial hepatectomy followed 9 days later (when they were 70–90 g. body weight) by the administration of a single oral dose of retrorsine, 30 mg./kg. body weight.

### *I. Experiments Involving Rats Given Retrorsine and Whole Body Irradiation*

#### *A. Lesions found in rats which died* before *whole body irradiation (WBR)*

Nineteen of the 50 rats given a single dose of retrorsine died during the first three months after its administration and before receiving WBR. Ascites, pleural effusions and gastro-intestinal haemorrhages were observed in the animals dying in the early stages following retrorsine. Nine out of these 19 rats were examined histologically.

The lesions in the livers of these rats were very similar to those described previously by Schoental and Magee (1959). Areas of haemorrhage, zonal necrosis and interstitial cellular infiltration were present in the early stages. From about 4 weeks after administration of the alkaloid the typical, greatly enlarged liver parenchymal cells were present, frequently occurring in discrete foci. By two months it was possible to detect very early fibrotic changes. In 2 rats ectopic bone was present in the livers (Fig. 1 and 2).

In addition, there appeared to be early necrotising vascular changes in the lung and heart which suggested a generalised acute inflammatory vascular process. In the lung of one rat the changes were seen in the large branches of the pulmonary artery and consisted of an acute necrotising arteritis with fibrinoid change and cellular infiltration of the vessel wall (Fig. 3). In another rat which died 40 days after retrorsine focal areas of myocardial necrosis were present with an associated acute inflammatory response (Fig. 4).

*B. Lesions found in rats which died* after *whole body irradiation.*

Nine of the 31 rats given retrorsine and whole body irradiation showed demonstrable evidence of liver fibrosis in varying degrees. Liver lesions were present in all these rats, and were similar to those described by Schoental and Magee (1959) for corresponding survival times of the animals. Two rats died 10 days after WBR from extensive gastro-intestinal haemorrhage, two more were lost about 4 months later ; one due to bronchopneumonia, the other due to middle ear infection, and two more rats were killed due to middle ear infection about 11½ months after retrorsine. In one rat, killed at 24 weeks after retrorsine, there was evidence of a healing arteritic lesion in an interlobar artery of the kidney (Fig. 5).

A wide variety of tumours arising in several different organs was observed among the 25 rats which survived more than a year after the alkaloid. These are listed in Table I together with the times of the death of the animals.

TABLE I.—*Tumours Found in Rats Which Survived More Than One Year, in the Four Experimental Groups* (*number of survivors in brackets*)

I : Retrorsine and whole body irradiation (25) ; II : Retrorsine (29) ; III : Whole body irradiation (6) ; IV : Partial hepatectomy and retrorsine (9).

| Type of tumour | I | | II | | III | | IV | |
|---|---|---|---|---|---|---|---|---|
| | Number of tumours | Time of death (months) | Number of tumours | Time of death (months) | Number of tumours | Time of death (months) | Number of tumours | Time of death (months) |
| Hepatomata | 5 | 15, 15, 21, 23, 23 | 5 | 13, 26, 29, 29, 29 | — | — | 2 | 19, 19 |
| Carcinoma of liver (+ pulmonary metastases) | 1 | 23 | — | — | — | — | — | — |
| Mammary tumours | 5 | 15, 15, 16, 21, 21 | 1 | 27 | — | — | — | — |
| Renal carcinoma | 2 | 20, 23 | — | — | — | — | — | — |
| Carcinoma of lung | 1 | 14 | 1 | 11 | — | — | — | — |
| Carcinoma of colon | 1 | 20 | — | — | — | — | — | — |
| Haemangio-endothelioma of spleen | 1 | 14 | 1 | 29 | — | — | — | — |
| Osteosarcoma of humerus | 1 | 21 | — | — | — | — | — | — |
| Leukaemia | 1 | 16 | — | — | 2 | 15, 18 | — | — |
| Spindle cell tumour of neck | 1 | 20 | — | — | — | — | — | — |
| Osteosarcoma of sternum | — | — | — | — | 1 | 19 | — | — |
| Carcinoma of uterus | — | — | 1 | 27 | — | — | — | — |
| Retro-peritoneal sarcoma | — | — | 1 | 18 | — | — | — | — |
| Squamous cell carcinoma of jaw | — | — | 1 | 24 | — | — | 1 | 17 |
| Renal adenoma | — | — | — | — | 1 | 15 | — | — |

Five of these rats had lesions in the liver classified as hepatomata. In the present context these have been defined as circumscribed areas of liver cells with atypical architectural arrangement. Very often collections of swollen and ballooned liver cells were observed. There were also associated cystic and ectatic changes of the biliary system. Occasionally the hepatomata showed angiomatous changes within them. Mitoses were infrequent.

The liver from the animal which showed the malignant changes was quite variable in pattern but there was virtually no normal liver structure to be seen (Fig. 6). Large areas of fatty change with marked dilatation of the bile ducts were present and many foci of large swollen cells with abundant cytoplasm. There was some evidence of early cirrhosis. At the free edge of the liver was a large well-differentiated hepato-cellular carcinoma (Fig. 7, 8, 9) and similarly well-differentiated metastases were present in the lungs (Fig. 10). The testis showed marked tubular atrophy and the absence of spermatogonial elements.

The mammary tumours, which in this series were in males, frequently showed structural changes of malignancy and in one case there was evidence of pulmonary metastasis. Compared with the histological structure of other radiation-induced mammary tumours there seemed to be a disproportionate degree of anaplasia in their structure. Whether this is connected in any way with some hormonal factor associated with liver impairment is diffcult to say.

The two renal tumours had a fairly typical structure, one being of the " clear cell " type and the other a papillary type (Fig. 11, 12).

Chronic renal disease of moderate to severe degree was observed in several of the experimental and control animals. In all cases these changes were observed in rats which were at least 15 months of age or older.

The carcinoma of the lung found in one rat of this group was of a squamous cell type (Fig. 13) and was associated with marked bronchiectatic changes with tumour emboli in the pulmonary vessels.

The carcinoma of the colon was a polypoid tumour ; there was no evidence of liver metastasis (Fig. 14).

The haemangio-endothelioma of the spleen was a small localised tumour which on histological examination showed several blood-filled sinusoidal spaces lined by enlarged endothelial cells showing frequent mitosis (Fig. 15, 16).

The osteosarcoma of the left shoulder region was too large to determine precisely from which part of the bone it arose but radiographic evidence indicated that it was arising from the upper or growing end of the humerus (Fig. 17). The radiograph showed a lifting-up and thickening of the periosteum. Microscopic examination showed a well-differentiated osteosarcoma with metastases to the scalp, liver, adrenal and lung (Fig. 18).

The leukaemia appeared on histological evidence to be of an acute myelogenous type.

A spindle cell tumour was present in the neck of the same rat which developed a renal carcinoma. Although this tumour is described as a " spindle cell " tumour, many of the cells in fact resembled osteoblasts and there was evidence of bone formation and calcification in the tumour.

### *II. Rats Given a Single Dose of Retrorsine*

Of the 95 rats given a single dose of retrorsine, 29 survived for more than a year, with a mean survival time of 23 months.

The liver lesions were similar to those described previously by Schoental and Magee (1959). Deposition of fibrous tissue in the liver and biliary ectasia were present in about one fifth of the animals. Hepatomata were observed in 5 rats, the mean survival time of which was 25 months. In one rat killed after 29 months an especially large liver tumour was present. The tumour (Fig. 19) lay in the right lobe of the liver and measured $5 \times 5 \times 2{\cdot}5$ cm. A small nodule was also present in the left lobe. The whole liver weighed 48 g. which represented about 12 per cent of the total body weight. Microscopically, the tumour was benign, its appearance consisting of abnormally orientated liver cords of uniform pattern but with no evidence of increased mitotic activity or metastasis (Fig. 20).

One other rat in which a hepatoma was present was also found to have a large, spherical tumour of the spleen measuring approximately 3 cm. in diameter (Fig. 21). The tumour was dusky bluish red in colour with a smooth surface and on microscopical examination consisted of closely packed cords of cells, the picture being consistent with that of a haemangio-endothelioma of the spleen (Fig. 22). Twelve of the rats in this group were females. One of these was killed at 27 months

---

### EXPLANATION OF PLATES

FIG. 1.—Liver of a rat which died 45 days after retrorsine. Focus of ectopic bone with myeloid metaplasia. H. and E. ×70.

FIG. 2.—Liver of a rat which died 110 days after retrorsine. Note the presence of ectopic bone. H. and E. ×70.

FIG. 3.—Large branch of a pulmonary artery in a rat which died 1 month after retrorsine. Note the presence of an acute necrotising arteritis with fibrinoid material present. Marked perivascular cellular infiltration. H. and E. ×70.

FIG. 4.—Acute focal myocarditis with muscle degeneration and thrombus present in a small coronary arteriole, 40 days after retrorsine. H. and E. ×70.

FIG. 5.—Arteritic lesion in the kidney of a rat killed 5½ months after retrorsine and WBR. H. and E. ×315.

FIG. 6.—The appearance at post mortem of the rat in which a malignant liver tumour was present 23 months after retrorsine and WBR. Note the presence of tumour nodules in all the visible lobes with a metastatic nodule on the inferior aspect of the left lower lobe of the lung.

FIG. 7.— Low power view of one of the liver nodules shown in Fig. 6. Note the papillary structure of the tumour together with a varied picture in the rest of the section. H. and E. ×4.

FIG. 8.—Another area of the same tumour as in Fig. 6 showing an area of fatty change adjacent to a hepatomatous area. H. and E. ×40.

FIG. 9.—Another field from the same tumour showing the changes of a hepato-cellular carcinoma. H. and E. ×280.

FIG. 10.—A pulmonary metastasis from the tumour depicted in Fig. 6. H. and E. ×40.

FIG. 11.—A well circumscribed renal carcinoma at the pole of the kidney with areas of haemorrhage and necrosis. H. and E. ×5.

FIG. 12.—The microscopic appearance of the renal tumour illustrated in Fig. 11. The structure resembles that of a clear-cell carcinoma with several mitotic figures present. H. and E. ×280.

FIG. 13.—Part of a squamous cell carcinoma present in the lung of a rat at 14 months. H. and E. ×80.

FIG. 14.—Three pedunculated tumours of the colon. Microscopically, the appearance was that of an adenocarcinoma. ×1.

FIG. 15.—Section of the spleen of a rat at 14 months after retrorsine + WBR showing a tumour nodule. H. and E. ×9.

FIG. 16.—Haemangio-endothelioma of the spleen of the rat illustrated in Fig. 15. Note the prominent endothelial cells with several mitotic figures. H. and E. ×230.

FIG. 17.—Radiograph of the left shoulder and skull of a rat 21 months after retrorsine + WBR showing an osteosarcoma of the left humerus.

FIG. 18.—Pulmonary metastasis present in the rat illustrated in Fig. 17. H. and E. ×43.

FIG. 19.—The liver of a rat 29 months after a single dose of retrorsine (but no WBR). The liver weighed 48 g. Note the central umbilication of the tumour and a second nodule present in the left lobe. ×0·5.

FIG. 20.—The edge of the tumour depicted in Fig. 19. The pattern is very regular and there is no evidence of malignant change. H. and E. ×47.

FIG. 21.—The gross appearance of the splenic tumour in a rat at 29 months after retrorsine. The maximum diameter was 3 cm.

FIG. 22.—The microscopic appearance of the tumour shown in Fig. 21. The normal follicular pattern is entirely absent and has been replaced by closely compressed sinusoids. The picture suggests a haemangio-endothelioma. H. and E. ×47.

1

2

3

4

5

6

7

8

9

10

11

12

13

Schoental and Bensted.

14

15

16

17

18

19

20

21

22

Schoental and Bensted.

and found to have two tumours, namely a cystic mammary tumour and a carcinoma of the uterus. The liver was not significantly changed.

A squamous cell carcinoma of jaw was found in one rat and in another animal a squamous cell carcinoma of the lung was present. Chronic renal disease was observed in 6 rats, generally towards the end of the second year of life.

### *III. Rats Exposed to a Single Dose of Whole Body Irradiation,* 400 *r*

Among the six rats in this group there were two examples of diffuse leukaemic infiltration of the tissues at 15 and 18 months. In one of these rats the microscopic evidence suggested a myelogenous form of leukaemia.

The renal tumour found at 15 months in one of the rats had the microscopic appearance of an adenoma of foetal type. In another rat which died at 19 months an osteosarcoma of the sternum was present. In these rats the livers did not show the characteristic lesions seen in rats given the alkaloid.

Among the rats exposed to radiation in this group as well as in group I several instances of cataract were observed.

### *IV. Rats Given a Single Dose of Retrorsine Following Partial Hepatectomy*

Of the 9 rats which survived for more than a year after partial hepatectomy followed by retrorsine, 2 were found to have developed hepatomata at 19 months. One squamous cell carcinoma of the jaw was present in this group.

The liver lesions in the rats in this group were similar to those seen at corresponding times in rats given retrorsine alone. As a result of the hepatectomy there was some enlargement of the right liver lobes in these rats.

### DISCUSSION

The observation that a single dose of the pyrrolizidine alkaloids can induce in rats chronic liver lesions (Schoental and Magee, 1959) and even hepatomata as described in the present experiments, raises several problems. So far nothing is known about the biochemical lesions brought about by the interaction of such water-soluble compounds with tissues and why and how these set in motion a sequence of changes which can ultimately lead to liver tumours. The induction of tumours of other organs by a single dose of other water-soluble chemical compounds has also been reported ; e.g. stomach tumours by *N*-methyl-*N*-nitrosourethane (Schoental, 1960 ; Schoental and Magee, 1962) ; kidney tumours by dimethylnitrosamine (Magee and Barnes, 1962) and leukaemia by urethane given to newborn mice (Fiore-Donati *et al.*, 1961). The development of a variety of tumours following a single dose of ionising radiation is well known. Both these types of carcinogenic agents—chemical and physical—appear to interfere with some fundamental process(es) of the cell—probably related to mitotic division. It has been suggested that they may possibly interfere with various stages of the biosynthesis of a " mitotic hormone " (Schoental, 1963).

The changes observed in the livers of rats after pyrrolizidine alkaloids, especially the persistence of enlarged parenchymal cells, morphologically easily recognisable from the normal sized ones, and showing abnormal mitotic figures (Schoental and Magee, 1959), appear particularly favourable for the study of factors which may influence the induction of liver tumours. However, no clearcut co-carcinogenic agents have as yet come to light in hepatic carcinogenesis. The reported use of partial hepatectomy following feeding with dimethylamino-azobenzene (Glinos, Bucher and Aub, 1951), or preceding or following the feeding with acetylaminofluorene (Laws, 1959) gave rather equivocal results. The same is true for the effects of whole body irradiation used in conjunction with azo-dye feeding (Hoch-Ligeti, 1949 ; Kato *et al.*, 1959 ; Williams, Young and Moore, 1951).

Co-carcinogenic action can best be demonstrated when sub-optimal doses of carcinogens are used. Supplementation of an " optimal " dose of a carcinogen, whether by the same agent or by a co-carcinogen may lead to more severe cell injury, atrophy of the affected organ and possibly to premature death of the animal, before tumours have time to appear. Thus, as a result of excessive dosing of rats with a single agent (a hepatotoxic pyrrolizidine alkaloid, heliotrine, given repeatedly three times weekly, 0·1 of a $LD_{50}$ per dose), the animals died with liver atrophy and no tumours (Bull and Dick, 1960). The experiments of Lacassagne and Hurst (1962) who used two agents, illustrate the point particularly well. These authors exposed only certain liver lobes to X-rays, shielding the remainder of the liver and the body of the rats ; one month later some of the animals were given a diet containing 0·06 per cent of *p*-dimethylaminoazobenzene. Atrophy was observed of the liver lobes which had been exposed to high doses of radiation, 1500 r and 3000 r, and compensatory hypertrophy of the non-irradiated parts of the livers. Neoplastic changes due to *p*-dimethylaminoazobenzene were mostly confined to the latter.

The purpose of the present experiments was to explore whether the development of primary liver tumours after a single dose of retrorsine might be affected by preceding hepatectomy, or by subsequent whole body irradiation. Hepatectomy *per se* has not been reported to cause tumours. Its effects, if positive, could thus be considered as co-carcinogenic. However, ionising radiation is itself a known carcinogenic agent and it was of interest to know whether its effects on the liver lesions could be regarded as (a) additive, (b) synergistic, or (c) inhibitory. The dose of 400 r whole body irradiation would not be expected to cause excessive damage to the liver.

In the present X-ray experiments a wide spectrum of tumours was observed. It is evident that many of them are merely the result of a single dose of whole body irradiation, and the types and number of tumours produced are consistent with the experience of other observers.

Mammary tumours are one of the most common tumours to follow whole body irradiation in female rats and not uncommonly in males (Binhammer *et al.*, 1957 ; Lamson *et al.*, 1957 ; Cronkite *et al.*, 1960). The other types of tumours have all been described following whole body irradiation, with the exception of malignant, metastasising liver tumours.

The liver is generally regarded as being a relatively resistant organ to radiation carcinogenesis. However, an important factor which must be considered is the effect of local as opposed to whole body irradiation. Weinbren, Fitshen and Cohen (1960) gave 5000 r locally to livers of rats and although they described frequent mitotic abnormalities, they did not mention tumour formation in rats which were killed a year after the irradiation. Furth, Upton and Kimball (1959) observed a 6 per cent incidence of hepatomata in mice after exposure to whole body X-radiation and 20 per cent incidence following 400 r radiation of the abdomen (including the liver) alone. No malignant tumours of the liver were reported. This observation is in agreement with the conclusion of Furth and Lorenz (1954) that whilst the endothelial cells may be susceptible to the carcinogenic effect of radiation there was no evidence to suggest that the parenchymal cells were similarly susceptible.

Among the 6 rats exposed to 400 r whole body radiation alone, the livers did not show the characteristic changes seen in animals given retrorsine. One of these rats developed an osteosarcoma of the sternum, one a renal adenoma and two had leukaemia.

In the present experiments, the number of liver tumours, both benign and malignant, is small, and a comparison of the incidence in the various groups is rather difficult because of the variation in the survival times of the animals. If the latent period of 23 months for the development of the liver carcinoma is to be taken as any sort of a guide for the development of malignant liver tumours, then it may reasonably be argued that there might well have been a greater yield of these tumours if there had not been a number of early deaths from other tumours which had clearly been produced by the effects of whole body irradiation. If a more certain relation between radiation and the effects of the alkaloid is to be established, then it seems that the radiation must be given locally to the abdomen or directly to the liver so that the carcinogenic effects of whole body radiation can be eliminated and thus permit a reasonable expectation of life for the presumed liver tumours to develop. Another alternative way of delivering an appreciable dose of radiation directly to the liver without affecting other organs would be to make use of an isotope such as radioactive gold which is removed from the blood stream by the reticulo-endothelial system and which has been used by Upton, Furth and Bennett (1956) to produce hepatomata in mice.

As with many hepatotoxic agents, the degree of susceptibility depends to a considerable extent on the nutritional status of the animal (Drill, 1952). In a similar fashion the changes in the liver following whole body radiation depend also on the animal's state of nutrition. White *et al.* (1955) showed that Sprague–Dawley rats fed on a low protein diet developed cirrhosis of the liver in 53 per cent of cases after 500 r WBR. In the present experiments, although the rats were on a " normal " diet there might have been some metabolic disturbance as judged by their lower body weight. Yet the liver lesions and the degree of fibrotic changes did not appear to differ strikingly in the 3 groups of rats which had the alkaloid. Very much larger numbers of animals would be required to permit statistical evaluation of the results, in view of the great difference in response of individual rats to the alkaloid.

Of especial interest are the pulmonary and cardio-vascular lesions which were observed in the rats which died within the first 3 months after receiving the alkaloid. Recently Lalich and Merkow (1961) observed pulmonary arteritis and myocarditis among rats which died after 3–5 weeks feeding with seeds of *C. spectabilis*, known to contain pyrrolizidine alkaloids. The authors suspected the presence of an additional angiotoxin in the seeds. However, in view of the presence of similar lesions in some of our rats which were given a single dose of a pure alkaloid, retrorsine, it is obvious that such lesions are one of the manifestations of pyrrolizidine alkaloids. The evolution of these lesions after administration of other pyrrolizidine alkaloids, especially fulvine, was studied in some detail, in view of their role in the " veno-occlusive " disease of Jamaica (Barnes, Magee and Schoental, to be published).

Some of the earlier workers (Davidson, 1935 ; Rosenfeld and Beath, 1945)

took the view that the liver lesion following pyrrolizidine alkaloids was primarily a vascular one. In view of the presence of acute necrotising vascular lesions in other organs in very young rats, it may well be that these alkaloids do indeed produce vascular changes but of a more generalised nature rather than being merely confined to the liver.

As far as the pulmonary changes are concerned, a point of especial significance is the effect which the healed arterial lesions may have on the right side of the heart and especially the hepatic veins. This could be manifested by morphological changes in the vein such as fibrous thickening of the wall. However, these ideas must remain speculative until there is some experimental evidence that pulmonary hypertension does in fact exist in the animals which receive pyrrolizidine alkaloids.

Partial hepatectomy did not appear to have affected significantly the chronic liver lesions due to retrorsine. In this group the rats survived better after the dose of retrorsine (30 mg./kg.), probably due to the fact that these animals were slightly older than the other rats. Susceptibility of rats to this alkaloid falls surprisingly sharply with the age of the animal (Schoental, 1959). This effect is not so striking in the case of some other pyrrolizidine alkaloids, e.g. lasiocarpine.

The liver changes seen in our rats in the 3 groups given retrorsine, whether alone or combined with irradiation or hepatectomy, were similar to those described by Schoental and Magee (1959) for the respective survival times of the animals. In addition hepatomata were observed in a small number of rats. The incidence of hepatomata did not vary greatly in the three groups. The average times at which hepatomata were observed in the three groups were : 25 months after retrorsine alone, 20 months after retrorsine and WBR, and 19 months after hepatectomy and retrorsine. This difference might superficially suggest a co-carcinogenic effect of the second agent. However, the experiment in which treatment with retrorsine was combined with irradiation was terminated after 23 months, while the rats given only retrorsine were allowed to survive as long as possible. In general, tumours can be present in an animal for some time without obvious ill-health and this is especially the case with liver tumours. The liver carcinoma was found in a rat which was killed at 23 months when still in relatively good condition.

Although no strong evidence has been obtained in the present experiments to support the idea of a synergistic action of whole body radiation and the pyrrolizidine alkaloid, some suggestive evidence may be adduced in that a clearly malignant liver tumour was present in this series.

### SUMMARY

1. Rats which survived 100 days after a single oral dose of retrorsine (30 mg./kg.) were exposed to whole body irradiation (400 r) ; 25 of these rats survived more than a year after the alkaloid, and developed a variety of tumours at various sites. Primary liver tumours, one of which was a hepato-carcinoma with metastases in the lung, were present in six rats.

2. Among 29 rats which survived more than a year after a single oral dose of retrorsine (30 mg./kg.) and received no further treatment, five developed hepatomata.

3. Two hepatomata were present among 9 rats which were given a single oral dose of retrorsine (30 mg./kg.) following partial hepatectomy.

4. Tumours at various sites (but not of the liver) were present in 6 rats which survived more than one year after whole body irradiation (400 r).

5. Whole body irradiation or partial hepatectomy did not inhibit the development of chronic liver lesions and liver tumours caused by a single oral dose of retrorsine. On the basis of the present experiments there is no definite evidence of a synergistic action due to these agents.

We wish to thank Professor W. V. Mayneord, C.B.E., Professor L. F. Lamerton and Dr. J. M. Barnes, C.B.E., for their interest and help. Also Mr. R. F. Legg for the microphotographs and Mr. M. R. Greenwood for valuable technical assistance.

### REFERENCES

BERENBLUM, I.—(1954) *Cancer Res.*, **14**, 471.
BINHAMMER, R. T., FINERTY, J. C., SCHNEIDER, M. AND CUNNINGHAM, A. W. B.—(1957) *Radiat. Res.*, **6**, 399.
BULL, L. B. AND DICK, A. T.—(1960) *Aust. J. exp. Biol. med. Sci.*, **38**, 515.
CRONKITE, E. P., SHELLABARGER, C. J., BOND, V. P. AND LIPPINCOTT, S. W.—(1960) *Radiat. Res.*, **12**, 81.
DAVIDSON, J.—(1935) *J. Path. Bact.*, **40**, 285.
DRILL, V. A. (1952) *Pharmacol. Rev.*, **4**, 1.
FIORE-DONATI, L., CHIECO-BIANCHI, L., DE BENEDICTIS, G. AND MAIORANO, G.—(1961) *Nature, Lond.*, **190**, 278.
FURTH, J., UPTON, A. C. AND KIMBALL, A. W.—(1959) *Radiat. Res.*, Suppl. 1, 243.
*Idem* AND LORENZ, E. (1954) ‘ Radiation Biology ’, edited by Hollander, A., Vol. I, Pt. 2., London (McGraw-Hill), p. 1184.
GLINOS, A. D., BUCHER, N. L. R. AND AUB, J. C.—(1951) *J. exp. Med.*, **93**, 313.
HOCH-LIGETI, C. (1949) *Brit. J. Cancer*, **3**, 562.
KATO, T., WATANABE, T., KAWASAKI, H., SUGIKATO, T., IBATA, H., HIROOKA, S., MIYAJI, T. AND KAWAI, K.—(1959) *Gann.*, Suppl. **49**, 168.
LACASSAGNE, A. AND HURST, L.—(1962) *Amer. J. Roentgenol.*, **87**, 536.
LALICH, J. J. AND MERKOW, L.—(1961) *Lab. Invest.*, **10**, 744.
LAMSON, B. G., MEEK, R. A. AND BENNETT, L. R.—(1957) *Arch. Path.*, **64**, 505.
LAWS, J. O.—(1959) *Brit. J. Cancer*, **13**, 669.
MAGEE, P. N. AND BARNES, J. M.—(1962) *J. Path. Bact.*, **84**, 19.
ROSENFELD, I. and BEATH, O. A.—(1945) *Amer. J. clin. Path.*, **15**, 407.
SCHOENTAL, R.—(1959) *J. Path. Bact.*, **77**, 485.—(1960) *Nature*, Lond., **188**, 420.—(1963) in ‘ Polycyclic Hydrocarbons ’, edited by Clar, E. New York (Acad. Press).
*Idem* AND HEAD, M. A.—(1957) *Brit. J. Cancer*, **11**, 535.
*Idem*, HEAD, M. A. AND PEACOCK, P. R.—(1954) *Ibid.*, **8**, 458.
*Idem* AND MAGEE, P. N.—(1957) *J. Path. Bact.*, **74**, 305.—(1959) *Ibid.*, **78**, 471.—(1962) *Brit. J. Cancer*, **16**, 92.
UPTON, A. C., FURTH, J. AND BENNETT, W. T., Jr.—(1956) *Cancer Res.*, **16**, 211.
WEINBREN, K., FITSHEN, W. AND COHEN, M.—(1960) *Brit. J. Radiol.*, **33**, 419.
WHITE, J., CONGDON, C. C., DAVID, P. W. AND ALLY, M. S.—(1955) *J. nat. Cancer. Inst.*, **15**, 1155.
WILLIAMS, G. Z., YOUNG, N. F. AND MOORE, J. P.—(1951) *Cancer Res.*, **11**, 289.